# Expression of Spermine Oxidase Is Associated with Colorectal Carcinogenesis and Prognosis of Patients

**DOI:** 10.3390/biomedicines10030626

**Published:** 2022-03-08

**Authors:** Sooyoun Kim, Doyeon Kim, Sanghyun Roh, Inpyo Hong, Hyeongjoo Kim, Tae Sung Ahn, Dong Hyun Kang, Moon Soo Lee, Moo-Jun Baek, Hyoung Jong Kwak, Chang-Jin Kim, Dongjun Jeong

**Affiliations:** 1 Department of Pathology, College of Medicine, Soonchunhyang University, 31 Soonchunhyang 6 gil, Dongnam-gu, Cheonan 31151, Chungcheongnam-do, Korea; sooy.kim@sch.ac.kr (S.K.); 20217098@sch.ac.kr (S.R.); 2 Soonchunhyang Medical Science Research Institute, College of Medicine, Soonchunhyang University, 31 soonchunhyang 6 gil, Dongnam-gu, Cheonan 31151, Chungcheongnam-do, Korea; ehdus0914@naver.com (D.K.); hip0725@sch.ac.kr (I.H.); khj@sch.ac.kr (H.K.); 3 Department of Surgery, College of Medicine, Soonchunhyang University, 31 Soonchunhyang 6 gil, Dongnam-gu, Cheonan 31151, Chungcheongnam-do, Korea; eyetoeye@schmc.ac.kr (T.S.A.); c100048@schmc.ac.kr (D.H.K.); msslee@schmc.ac.kr (M.S.L.); ssurge@sch.ac.kr (M.-J.B.); 4 Research Institute of Clinical Medicine, Woori Madi Medical Center, 111 Baekjedae-ro, Wansan-gu, Jeonju 55082, Jeollabuk-do, Korea; k-h-jong@hanmail.net (H.J.K.); mountain48@hanmail.net (C.-J.K.)

**Keywords:** colorectal cancer (CRC), SMOX, prognostic marker, therapeutic target

## Abstract

Uncovering tumor markers of colorectal cancer is important for the early detection and prognosis of the patients. Spermine oxidase (SMOX) is upregulated in various cancers. The present study aims to explore the biologic function and expression patterns of SMOX in colorectal cancer (CRC), the third most common type of cancer worldwide. We used quantitative real-time PCR, Western blot, and in vitro functional studies in four CRC cell lines knocked down by SMOX siRNA and immunohistochemistry in 350 cases of CRC tissues. The results showed that SMOX was overexpressed in CRC cell lines and clinical samples. SMOX overexpression in tumor tissues was an independent prognostic factor, worsening overall survival (*p* = 0.001). The knock-down of SMOX inhibited CRC cell proliferation, invasion, and soft agar colony formation, uncovering its carcinogenic functions. This study indicated that SMOX overexpression could be an important oncogene in CRC and might serve as a valuable prognostic marker and potential therapeutic target for CRC.

## 1. Introduction

Colorectal cancer (CRC) is the third most frequent cancer and the second leading cause of death worldwide [1]. In the Republic of Korea, CRC is the third most common cancer and third leading cause of cancer-related death [2]. The survival of colorectal cancer patients is different depending on the cancer recurrence and metastasis. Furthermore, the survival rate of CRC patients with localized colorectal cancer is 95.3%. If the cancer has only spread to the surrounding tissues, organs, and/or regional nodes, the survival rate is 82.6%; however, if a patient has distant metastatic CRC, the survival rate decreases to 20.2% [2]. In addition, the recurrence rate after curative resection for colorectal cancer has been reported to range from 30% to 50%. The 5 year overall survival rates for recurrences involving localized and metastasized cases were 78.8% and 34.7%, respectively [3]. The prediction of CRC recurrence is especially crucial because it decides if adjuvant chemotherapy or targeted molecular therapy should be administered. However, risk assessment of CRC is not easy because of the absence of reliable prognostic molecular biomarkers.

By analyzing the molecular basis of CRC, several biomarkers that are significantly correlated with the characteristics and progression of CRC have been identified [4,5,6,7,8,9,10]. The prognostic markers can increase the sensitivity of conventional treatments for CRC [11]. In particular, the identification of prognostic biomarkers could help oncologists to optimize therapies that are identified as challenges in the treatment of CRC patients. Despite the great progress that has been made in the development of novel therapeutic methods, the mortality of CRC patients remains relatively high due to the lack of prognostic biomarkers for CRC.

Spermine oxidase (SMOX) was recently revealed to be involved in polyamine (PA) metabolism and performs a main role in the catabolism of highly modulated mammalian PAs. SMOX oxidizes spermine to spermidine and makes reactive oxygen species in that process [12]. Oxidative-stress-induced reactive oxygen species result in the apoptosis of epithelial cells but also increase DNA damage, which increases the risk of tumorigenesis [13,14]. Furthermore, SMOX involves several pathological conditions, including cancer, drug response, and the response to stressful stimuli. Recent studies have reported that high expression of SMOX is related to gastric [15] and hepatocellular carcinomas [16]. Therefore, we studied the correlation of SMOX expression and CRC.

In this study, we investigated the role of SMOX protein in CRC progression and evaluated the effects of inhibiting SMOX expression using an siRNA-based technique on the proliferation, migration, invasiveness, and colony-forming ability of CRC cells. The results presented below highlight the potential importance of SMOX as a prognostic marker and a therapeutic strategy for CRC.

## 2. Materials and Methods

### 2.1. Cell Lines and Culture Conditions

The human CRC cell lines HCT116, HT29, SW480, and SW620 were purchased from the Korean Cell Line Bank (KCLB, Seoul, Korea). Each cell line was grown at 37 °C in Roswell Park Memorial Institute (RPMI) 1640 medium (Hyclone, Logan, UT, USA) supplemented with L-glutamine, heat-inactivated 10% fetal bovine serum (FBS, Corning, Corning, NY, USA), and 1% penicillin-streptomycin (Corning, Corning, NY, USA) in a fully humidified incubator containing 5% CO_2_.

### 2.2. Silencing of SMOX by siRNA

Specific SMOX siRNA (specific target sequence, 5′-GUUGAGGAAUUCAGCGAUU-3’) was purchased from Bioneer (Daejeon, South Korea). Approximately 1 × 10^5^ colorectal cancer cells were seeded in 6-well plates, incubated overnight, and then replaced with serum-free medium. Subsequently, the cells were transfected with 10 μL (5 nM) of SMOX siRNA and 20 μL of Hiperfect reagent (Qiagen, Hilden, Germany, #301705) in 70 μL of Opti-MEM^®^ medium and 900 μL of serum-free medium. After 36 h, the transfected cells were harvested and cells were isolated to obtain the RNA and protein. Then, these were evaluated for SMOX knockdown. The experiments were performed in triplicate.

### 2.3. Reverse Transcription Polymerase Chain Reaction (RT-PCR)

Total RNA was extracted from the cultured cells using the RiboEx reagent (GeneAll, Seoul, Korea). The following oligonucleotide primers pairs were used: SMOX forward primer, 5′- CCT GAG TGC AAC AGC CTA CA-3′; SMOX reverse primer, 5′-ATCGGGGGTTCTGTGAACTG-3′; GAPDH forward primer, 5′-CTTAGCACCCCTGGCCAAG-3′; and GAPDH reverse primer, 5′- GATGTTCTGGAGAGCCCCG-3′. The PCR products were electrophoresed in a 2% agarose gel at 100 V for 30 min and visualized by FluoroBox (CELLGENTEK, Daejeon, Korea). All analyses were performed in triplicate.

### 2.4. Western Blot Analysis

The total protein was extracted from cells with high purity through a PRO-PREP™ Protein Extraction Solution (iNtRON, Seongnam, Korea). The total protein concentration was determined by a bicinchoninic acid (BCA) assay. Next, 30 µg of the same protein per lane was separated by 10% SDS-PAGE, which separated the different molecular weights and transferred them onto nitrocellulose membranes (Merck Millipore, Burlington, US-MA, USA). The transferred membrane was blocked in 5% bovine serum albumin (BSA) in Tris-buffered saline containing 0.05% Tween-20 for 1 h. β-Actin was used as a loading control. The anti-human primary rabbit polyclonal SMOX antibody (Proteintech, Rosemont, IL, USA) was diluted at 1:500, and the membrane was subsequently incubated with the antibody in BSA at 4 °C overnight. 

### 2.5. Cell Proliferation Assay

siRNA-treated and untreated cells were uniformly dispensed at 1 × 10^4^ cells per 96 well plates, and incubated for 24 h, 48 h, and 72 h in 5% CO_2_ at 37 °C. Next, 10 μL (5 mg/mL) of EZ-CYTOX (DOGEN, Seoul, Korea) was added, followed by a 2 h incubation. The absorbance was measured at 450 nm using an xMark Microplate Absorbance Spectrophotometer (Bio-Rad, Hercules, US-CA, USA) to measure the cell proliferation and vitality. Statistical analyses were performed using the measured absorbance values to calculate the statistical significance.

### 2.6. Cell Invasion and Migration Assays

Using the transwell system, cell invasion and migration ability were confirmed. In particular, the invasion assay was used after coating the growth-factor-reduced Matrigel in the insert. After the colorectal cancer cells were diluted to 5 × 10^5^ cells with serum-free medium, they were added to the insert of transwell system and cultured for 48 h. Next, 3.7% paraformaldehyde was used to fix the transwell, and crystal violet was used to stain the cells. The number of cells in the membrane under the insert of the transwell was counted using an inverted microscope.

### 2.7. Semisolid Agar Colony-Forming Assay

The plates were coated with base agar (0.5% agar in growth medium with FBS). Equal numbers of transfected cells and control cells were placed 5000 cells/well with 0.35% agar and overlaid on base agar in six-well plates. The cells were then incubated for two weeks. After, the cells were stained with crystal violet (dilution for 0.05% in PBS) and then counted.

### 2.8. Immunohistochemistry (IHC)

CRC tissue was obtained from 350 patients at Soonchunhyang University Cheonan Hospital who underwent surgery prior to medical treatment and were histopathologically confirmed to have CRC. The patient data is presented in Table 1. This study was approved by the Ethics Committee of Soonchunhyang University, Cheonan Hospital (2018-07-061-006).

Paraffin-embedded blocks of CRC tissues were cut into 5 µm thick sections, and the tissue sections were placed on microscope slides. The slides were retrieved and incubated overnight at 4 °C with an anti-human SMOX antibody (1:100, Proteintech, Rosemont, IL, USA). After the slides were washed, the secondary antibody (Dako, Santa Clara, CA, USA; EnVision + System-HRP-labeled polymer anti-rabbit) reaction was performed at room temperature for 30 min. Finally, DAB (3-3-diaminobenzidine, Dako, Santa Clara, CA, USA) chromogenic substrate solution was used to visualize specific staining, and nuclei were stained with hematoxylin for counterstaining.

Immunohistochemical parameters were graded in a blinded manner by three independent investigators. They proceeded according to a semi-quantitative optical analysis and determined a score for each sample. The expression of SMOX in tumor cells was quantified by evaluating the percentage and intensity. Staining ratios were scored as 0 (0–5%), 1 (5–25%), 2 (25–50%), 3 (50–75%), and 4 (75–100%) points, and the staining intensity was scored as 0 for negative, 1 for weak staining, and 2 points for strong staining. The final score was calculated by multiplying the staining ratio score and the staining intensity score. Based on the final score, they were grouped into a group with low SMOX expression and a group with high SMOX expression (low-expression group scores <2; high-expression group scores ≥2, 2 was the mean value of the scores) by multiplying the percentage score by the intensity score.

### 2.9. Statistical Analysis

For statistical data processing, SPSS ver. 19.0 (IBM, Armonk, NY, USA) was used, and the relationships between the clinical factors and SMOX expression were analyzed using a chi-square test. Kaplan–Meier curves and Cox regression tests were used for survival rate analysis, and the log-rank test was used to compare survival rates. Statistical results were used to evaluate cases where the *p* value was less than 0.05.

## 3. Results

### 3.1. Validation of SMOX Expression and Transduction of siRNA in Colon Cancer Cell Lines 

**Figure 1:**
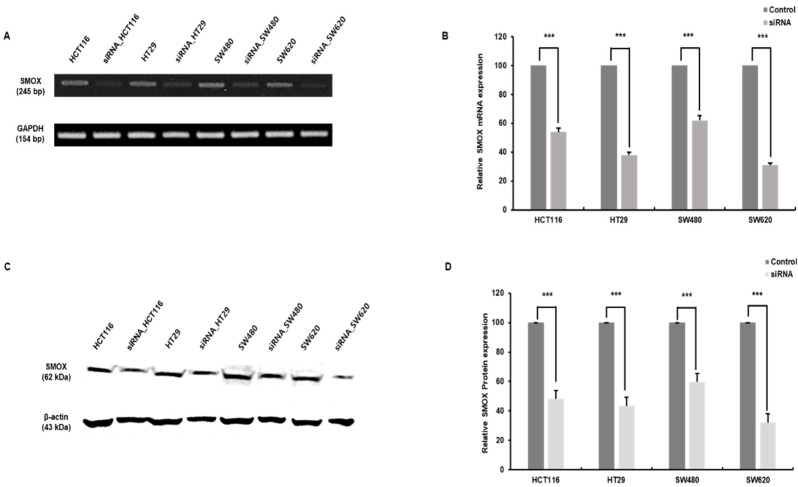
Expression of SMOX in colorectal cancer (CRC) cell lines and the effects of SMOX siRNA (small interfering RNA). SMOX expression and inhibition were confirmed by RT-PCR and immunoblot. (**A**) Screening by RT-PCR of SMOX in CRC cell lines and confirmation of SMOX inhibition compared with control cells. (**B**) Relative RNA expression of SMOX. The expression of SMOX siRNA-transfected cell lines was significantly lower than that in the control cell lines (*** p < 0.001). (**C**) Expression levels of SMOX and β-actin were determined by Western blotting in CRC cell lines. (**D**) Relative protein levels of SMOX. SMOX siRNA-transfected cell expression levels were lower than those in the control (*** p < 0.001).

We validated SMOX expression in four cell lines (HCT116, HT29, SW480, and SW620) in CRC and transfected them with siRNA to confirm the role of SMOX expression. Four CRC cell lines and transfected SMOX knockdown cells were subjected to RT-PCR that targeted human SMOX mRNA, and proteins were identified by Western blotting with specific targeting antibodies. Four siRNA-transfected CRC cell lines displayed significantly reduced SMOX expression levels when comparing mRNA levels (Figure 1A,B). The protein levels were confirmed in the control groups of the cell lines and were significantly decreased in the SMOX siRNA-treated cell lines (Figure 1C,D).

### 3.2. Proliferation Inhibition of Colon Cancer Cells by Downregulation of SMOX

**Figure 2:**
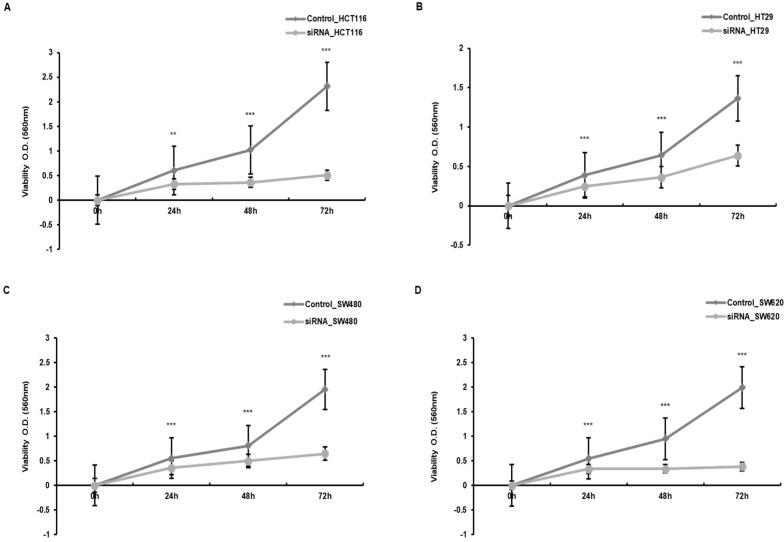
The comparison of the cell proliferation rate between control cells and siRNA-transfected cells in the CRC cell lines. (**A**) HCT116, (**B**) HT29, (**C**) SW480, and (**D**) SW620 (** *p* < 0.01, *** *p* < 0.001).

We used siRNA transduction to silence the expression of SMOX. The downregulation of SMOX has been observed to be significant based on previous results. To investigate the effect of SMOX expression on the proliferation of CRC cells, we performed a WST-1 analysis. When comparing CRC cells and cells whose expression of SMOX was downregulated, the rate of growth and proliferation was significantly lower than that of the control group (Figure 2).

### 3.3. Downregulation of SMOX Decreases Metastatic Capacity in Colorectal Cancer

**Figure 3:**
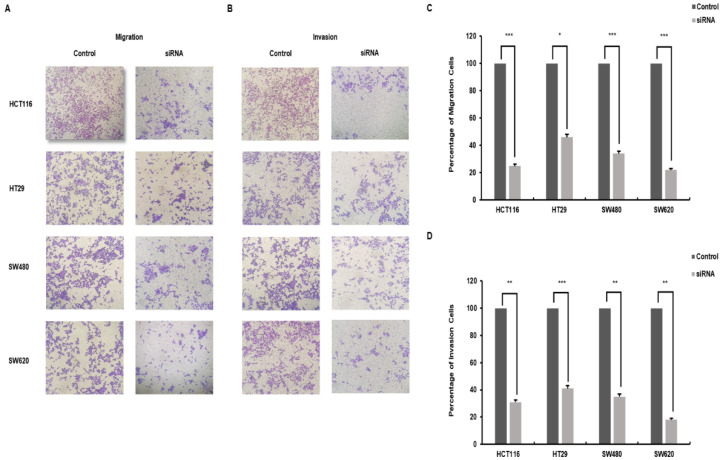
The results of the transwell assay regarding metastasis. Reduced expression of SMOX inhibits the migration and invasion abilities of CRC cells. The result of the chamber migration assay and invasion assay. Cells were visualized in violet color. (**A**) Migration of the siRNA treatment group was reduced in comparison with the control group. The images of the cells were on the bottom membrane (Original magnification ×40). (**B**) siRNA-treated cells were less numerous than control cells. The images of the cells were on the bottom membrane (Original magnification ×40). (**C**) The graph indicates the percentage of cells and the results of the chamber migration assay (HCT116, 35%; HT29, 46%; SW480, 34%; and SW620, 22%) (* *p* < 0.05, *** *p* < 0.001). (**D**) The graph expresses the percentage of cells in the chamber invasion assay (HCT116, 31%; HT29, 41%; SW480, 35%; and SW620, 18%) (** *p* < 0.01, *** *p* < 0.001).

To further characterize the role of SMOX expression in cancer progression, a transwell chamber assay was performed to investigate the migration and invasion of CRC cells. As shown in Figure 3A,B, CRC cells whose SMOX expression was downregulated significantly resulted in decreased cell migration and invasion compared to control cells. In the migration assay, the group treated with SMOX target siRNA showed a significant decrease in the migration of cells to the lower chamber compared to the control group (68.2%) (*p* < 0.05). The four reduced proportions were as follows: HCT116, 75%; HT29, 54%; SW480, 66%; and SW620, 78% (Figure 3C). In the invasion assay, SMOX-downregulated cells showed a significant decrease compared to the control (68.7%) (*p* < 0.05). The percentage reduction for each cell was as follows: HCT116, 69%; HT29, 59%; SW480, 65%; and SW620, 82% (Figure 3D). In the metastasis cell SW620, compared to the primary cell SW480, it was confirmed that the invasion capacity had an increased reduction due to the downregulation of SMOX expression. 

### 3.4. Independent Growth of Colorectal Cancer Cells Is Reduced by the Expression of SMOX

**Figure 4:**
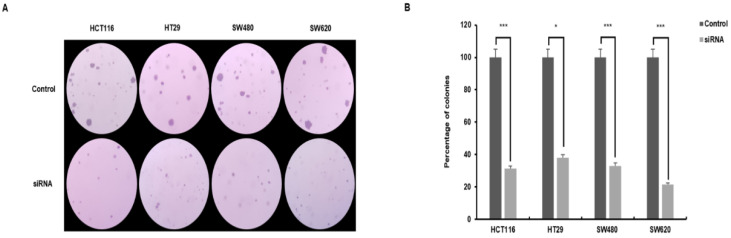
The result of thee semisolid agar colony-forming assay. Anchorage-independent growth in soft agar was examined with control cells and cells that displayed downregulation of SMOX expression. The numbers of colonies were measured after 14 days. Colony formation in the siRNA-treated group was reduced compared to that in the control group (HCT116, 31%; HT29, 38%; SW480, 33%; and SW620, 21%). Colonies were visualized in violet color. (**A**) Comparison of the growth of colonies with control cells and siRNA-treated cells (Original magnification ×40). (**B**) The graph indicates the number of colonies and the percentage difference between the control cells and siRNA-treated cells (* *p* < 0.05, *** *p* < 0.001).

An agar colony formation assay was performed to determine whether SMOX is required for anchorage-independent growth, a hallmark of oncogenic transformation. Anchorage and growth regulation of normal and viral transformed cells was observed. SMOX target siRNA-transfected cells showed a significant decrease in colony formation and efficiency compared with the control (Figure 4A). The number of colonies in the control and experimental groups of the four cell lines was as follows. HCT116 215 ± 5.69 and 68 ± 2.52; HT29 346 ± 45.74 and 132 ± 3.06; SW480 320 ± 15.87 and 106 ± 5.29; and SW620 212 ± 20.60 and 45 ± 2.08. The SMOX knockdown cells showed a reduction in colony formation efficiency of 31 ± 6.97% compared to control cells. The colony changes in the four cell lines were as follows. HCT116, 69% (*p* < 0.001); HT29, 62% (*p* < 0.05); SW480, 67% (*p* < 0.001); and SW620, 79% (*p* < 0.001) (Figure 4B). These results suggest an important relationship between SMOX silencing and anchorage-independent growth in CRC cells.

### 3.5. The Association of SMOX with a Poor Prognosis of Colorectal Cancer Patients 

**Figure 5:**
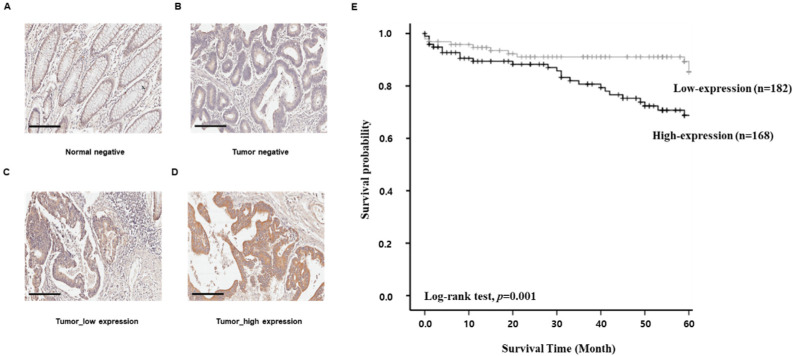
The result of IHC for SMOX expression in CRC (**A**–**D**). In total, 350 specimens were used, which were scored by multiplying the staining frequency and staining intensity. Low/High expression of SMOX was discovered in 182/168 CRC tissues. The Kaplan–Meier survival analysis was performed for all the sampled CRC patients, based on numerous variables and their expression of SMOX. SMOX proteins and nuclei were visualized by brown and violet, respectively. (**A**) Normal tissue without expression of SMOX (Original magnification ×200). (**B**) Cancer tissue without expression of SMOX (Original magnification ×200). (**C**) Cancer tissue with low expression of SMOX (Original magnification ×200). (**D**) Cancer tissue with high expression of SMOX (Original magnification ×200). (**E**) SMOX expression and overall survival of colon cancer patients were related. Following the expression of SMOX, patients were divided into two groups and the survival rate was determined using a Kaplan-Meier analysis (*p* = 0.001).

IHC was performed to assess the protein expression of SMOX in CRC specimens from patients, and a positive expression was found, mainly in the cell membrane, made evident by brown staining (Figure 5A–D). Overall, 350 specimens from CRC patients were used. A low expression of SMOX protein was detected in 182 CRC tissues (52%), whereas 168 CRC tissues (48%) exhibited a high expression of SMOX (Table 1). The associations between SMOX expression and CRC patient age, sex, pT and pN stage, metastasis, vascular or lymphatic invasion, and overall stage along with survival and death are summarized. Other pathological factors were not significantly associated with SMOX expression. 

We examined the relationship between SMOX expression and clinical outcomes. A high expression of SMOX resulted in shorter survival periods than those with a low expression of SMOX as determined by Kaplan–Meier survival assays (log-rank test, *p* = 0.001, Figure 5E). Univariate and multivariate analyses relating clinicopathologic factors to CRC patient prognosis were performed (Table 2). The univariate analysis showed that the following factors were significantly related to overall survival (stage, *p* = 0.01; pN stage, *p =* 0.016; metastasis, *p* = 0.038; and lymphatic invasion, *p* = 0.003). In addition, SMOX positivity was correlated with stage (*p* = 0.022) using a multivariate Cox regression analysis. These results indicate the close association of SMOX with the poor prognosis of patients with CRC.

## 4. Discussion

To understand cancer recurrence and metastasis as well as to prediction responses to cancer therapy or cancer progression it is important to characterize cancer heterogeneity. Sufficiently sensitive biomolecular markers for effective CRC diagnosis, prognosis, and therapeutic targets for treatment are still in demand to improve the excellence of clinical management. 

Natural PAs are polycationic amine derivatives. They play essential roles in cellular processes as cell cycle regulation, cell proliferation, differentiation, and tumor growth by interacting with DNA and chromatin [17]. SMOX, spermidine/spermine N1-acetyl transferase, and acetylpolyamine oxidase are essential for maintaining PA homeostasis in vertebrates [18]. They take part in drug response, apoptosis, and the pathogenesis of several cancer, such as hepatoma [16], gastric cancer [15], prostate cancer [19], neuroblastoma [20], uterine cervical cancer [21], lung cancer [22], and breast cancer [23]. The direct interplay between oncogenes and PA metabolism was first made obvious by the fact that ornithine decarboxylase (ODC), a PA, is a transcriptional target of the MYC oncogene [24]. Growth stimuli resulting in increased MYC expression lead to increased ODC1 mRNA, ODC protein, and activity; consequently, cells were stimulated to divide with the results of the increase in polyamines necessary for proliferation. Therefore, the MYC signaling pathway is one of the most important drivers of dysregulated polyamine metabolism-associated cancers. The aberrant c-Myc signaling pathway plays important roles in the carcinogenesis of CRC. Aberrant expression of c-Myc was observed in 70% of human cancers [25] and was elevated in 70–80% of CRC cases [26]. SMOX and c-Myc are similarly often overexpressed in CRC [27]. Aberrant PA metabolism in CRC is usually associated with chronic inflammatory mediators, such as C/EBP beta, rather than with the development of an infection by Bacteroides fragilis, for example. This depicts c-Myc and C/EBP beta as important key enzymes of the PA metabolic pathway in CRC.

In this study, we investigated the important cancer phenotypic role of SMOX expression in CRC cell lines. We found that SMOX was significantly upregulated in CRC cell lines. We applied the RNA interference technique to downregulate SMOX expression in four CRC cell lines (HCT116, HT29, SW480, and SW620) after validation of SMOX expression in these cell lines. We observed that SMOX knockdown suppressed cell proliferation and invasion in vitro. Importantly, knockdown of SMOX expression markedly decreased colony formation of the cells on semisolid agar, suggesting a carcinogenic effect of SMOX expression. SMOX expression was higher in CRC tissues than in normal adjacent non-tumorous tissues and was analyzed using clinicopathological parameters.

Therefore, the results clearly indicate that SMOX is involved in CRC cell migration, invasion, and metastasis. Furthermore, univariate and multivariate analyses indicated that high SMOX expression is an independent prognostic factor. 

SMOX is known to be an important enzyme that converts spermine to spermidine. High expression of spermine is reported to be a prognostic marker in CRC [28,29]. Therefore, high expression of SMOX in poor prognostic CRC in this study is reasonable result. However, the precise mechanisms of SMOX high expression in CRC need further studies.

In summary, our study is the first to demonstrate that SMOX plays an oncogenic role in the tumorigenesis of CRC in vitro. Furthermore, high SMOX expression is an independent prognostic factor for CRC and may serve as a future therapeutic target.

## Figures and Tables

**Table 1:** Comparison of clinicopathological factors and SMOX expression in patients with CRC.

Clinicopathological Factors	SMOX Expression		Total (N = 350)	*p* Value
	Low (N = 182)	High (N = 168)		
Age, years, mean (SD)	67 (12.8)	68 (12.6)	67 (12.7)	0.059
Gender, N (%)				0.04
M	85 (57.8)	62 (42.2)	147	
F	97 (47.8)	106 (52.2)	203	
pT, N (%)				0.197
pT1 and pT2	15 (62.5)	9 (37.5)	24	
pT3 and pT4	167 (51.2)	159 (48.8)	326	
pN, N (%)				0.296
pN0 and pN1	112 (50.7)	109 (49.3)	221	
pN2 and pN3	70 (54.3)	59 (45.7)	129	
Metastasis, N (%)				0.103
Negative	173 (53.1)	153 (46.9)	326	
Positive	9 (37.5)	15 (62.5)	24	
Vascular invasion, N (%)				0.115
Negative	157 (53.6)	136 (46.4)	293	
Positive	25 (43.9)	32 (56.1)	57	
Lymphatic invasion, N (%)				0.498
Negative	130 (52.2)	119 (47.8)	249	
Positive	52 (51.5)	49 (48.5)	101	
Stage, N (%)				0.195
Ⅰ and Ⅱ	99 (49.7)	100 (50.3)	199	
Ⅲ and Ⅳ	83 (55.0)	68 (45.0)	151	

**Table 2:** Cox regression analysis of the clinicopathological factors in CRC.

Clinicopathologic Factors	Variable	Univariate Analysis		Multivariate Analysis	
		Hazard Ratio (95%/CI)	*p* Value	Hazard Ratio (95%/CI)	*p* Value
Age	<60 yr vs. ≥60 yr	1.180 (0.621–2.240)	0.614	1.343 (0.683–2.642)	0.392
Gender	Male vs. Female	0.912 (0.481–1.729)	0.778	0.917 (0.464–1.814)	0.804
pT	T1-T2 vs. T3-T4	2.412 (0.855–6.805)	0.096	1.309 (0.424–4.044)	0.640
pN	N0 vs. ≥N1	2.189 (1.157–4.142)	0.016	0.440 (0.132–1.465)	0.181
Metastasis	M0 vs. ≥M1	2.718 (1.057–6.987)	0.038	0.877 (0.280–2.752)	0.823
Vascular invasion	Negative vs. Positive	1.630 (0.746–3.559)	0.221	0.926 (0.376–2.282)	0.888
Lymphatic invasion	Negative vs. Positive	2.654 (1.402–5.022)	0.003	1.694 (0.720–3.985)	0.227
Stage	Stage 1–2 vs. Stage 3–4	3.083 (1.574–6.037)	0.001	4.518 (1.239–16.479)	0.022
SMOX expression	Low vs. High	3.248 (1.579–6.682)	0.001	3.316 (1.523–7.222)	0.003

## Data Availability

All data necessary to support the reported results are present in the main text of the article.
